# Differences in the Prevalence of Non-Communicable Disease between Slum Dwellers and the General Population in a Large Urban Area in Brazil

**DOI:** 10.3390/tropicalmed2030047

**Published:** 2017-09-16

**Authors:** Robert E. Snyder, Jayant V. Rajan, Federico Costa, Helena C. A. V. Lima, Juan I. Calcagno, Ricardo D. Couto, Lee W. Riley, Mitermayer G. Reis, Albert I. Ko, Guilherme S. Ribeiro

**Affiliations:** 1 Division of Epidemiology, University of California, Berkeley, CA 94720, USA; robert.snyder@berkeley.edu; 2 Department of Medicine, University of California, San Francisco, CA 94143, USA; jayant.rajan@ucsf.edu; 3 Gonçalo Moniz Institute, Oswaldo Cruz Foundation, Brazilian Ministry of Health, Salvador 40170-115, Bahia, Brazil; fcosta2001@gmail.com (F.C.); helencris2006@gmail.com (H.C.A.V.L.); calcagnoji@gmail.com (J.I.C.); miter@bahia.fiocruz.br (M.G.R.); albert.ko@yale.edu (A.I.K.); 4 Institute of Collective Health, Federal University of Bahia, Salvador 40170-115, Bahia, Brazil; 5 Faculty of Pharmacy, Federal University of Bahia, Salvador 40170-115, Bahia, Brazil; rdc@ufba.br; 6 School of Medicine, Federal University of Bahia, Salvador 40170-115, Bahia, Brazil; 7 Division of Infectious Diseases and Vaccinology, University of California, Berkeley, Berkeley, CA 94720, USA; lwriley@berkeley.edu; 8 Department of the Epidemiology of Microbial Diseases, Yale University School of Public Health, New Haven, CT 06510, USA

**Keywords:** epidemiology, chronic illness, urban slum, inequality, favela

## Abstract

Residents of urban slums are at greater risk for disease than their non-slum dwelling urban counterparts. We sought to contrast the prevalences of selected non-communicable diseases (NCDs) between Brazilian adults living in a slum and the general population of the same city, by comparing the age and sex-standardized prevalences of selected NCDs from a 2010 survey in Pau da Lima, Salvador Brazil, with a 2010 national population-based telephone survey. NCD prevalences in both populations were similar for hypertension (23.6% (95% CI 20.9–26.4) and 22.9% (21.2–24.6), respectively) and for dyslipidemia (22.7% (19.8–25.5) and 21.5% (19.7–23.4)). Slum residents had higher prevalences of diabetes mellitus (10.1% (7.9–12.3)) and of overweight/obesity (46.5% (43.1–49.9)), compared to 5.2% (4.2–6.1) and 40.6% (38.5–42.8) of the general population in Salvador. Fourteen percent (14.5% (12.1–17.0)) of slum residents smoked cigarettes compared to 8.3% (7.1–9.5) of the general population in Salvador. The national telephone survey underestimated the prevalence of diabetes mellitus, overweight/obesity, and smoking in the slum population, likely in part due to differential sampling inside and outside of slums. Further research and targeted policies are needed to mitigate these inequalities, which could have significant economic and social impacts on slum residents and their communities.

## 1. Introduction

Over the course of the past 25 years, non-communicable diseases (NCDs)—including neoplasms, cardiovascular and circulatory diseases, chronic respiratory diseases, cirrhosis of the liver, digestive diseases, neurological disorders, mental and behavioral disorders, diabetes, and musculoskeletal disorders—have gradually displaced communicable diseases, maternal, neonatal, and nutritional disorders, and injuries, as the principal global causes of illness. In 1990, NCDs accounted for only 43% of global disability-adjusted life years (DALYs), whereas in 2013 they accounted for 54% of global DALYs [1,2]. Surveys producing these data rarely quantify or describe health disparities between populations living in the same geographic area, such as within the same city, and this is particularly true in urban slums.

The United Nations Human Settlements Program estimates that roughly 863 million people live in urban slums in low- and middle-income countries [3]. Slums are operationally defined by the United Nations as urban areas with overcrowding, poor sanitation infrastructure, limited access to safe water, and poor structural quality of housing [4]. Access to healthcare, which has a substantial impact on NCD outcomes [5], is also less available in these communities. These slum-defining phenomena synergistically interact to cause more NCDs and their sequelae in slum populations than in residents of the same city that do not live in slums [6]. Further, in part due to the persistence of environmental risk factors for communicable diseases in many of these communities, residents suffer a dual burden of communicable and non-communicable diseases. 

Existing surveys of NCDs and NCD risk factors in slums tend to focus on individual NCDs. Further, many studies done in urban areas do not disaggregate the population’s health data by slum and non-slums status to allow for the detection of intra-urban health disparities, or even sample from this population [7,8,9,10,11,12,13,14,15,16,17]. 

Brazil is the largest country in South America, with an estimated 45.7 million (28.0%) of its residents residing in slums [18]. It has undergone a substantial demographic (and epidemiologic) transition over the past 30 years, with an increase in NCD and relative decreases in the incidence of communicable disease [1,19,20,21]. Previous studies have not compared the burden of disease among Brazilians living in slums, colloquially referred to as *favelas*, to that in the general Brazilian population. Here we report the findings of a survey of the prevalences of NCDs and NCD risk factors among adults living in a *favela* in Salvador, Brazil and compare those prevalences with the prevalences of NCDs and NCD risk factors in the general population of the same city.

## 2. Materials and Methods

### 2.1. Study Site

In 2010, Salvador had the third largest population in Brazil (2,676,606 inhabitants), and the greatest proportion of its residents (33%) living in slums [22]. In 2003, the Centro de Pesquisas Gonçalo Moniz, Fundação Oswaldo Cruz (CPqGM-Fiocruz) established a study site in Pau da Lima, a *favela* in Salvador, to conduct surveillance for acute febrile illnesses and cohort studies of leptospirosis. Of the city’s estimated *favela* population of 882,204 people, 76,532 (8.7%) reside within Pau da Lima [23,24]. A detailed map and description of Pau da Lima can be found in Hagan et al. (2016) [25]. Briefly, it is an area on the periphery of Salvador highlighted by hills and valleys, which was a sparsely populated area of Atlantic rainforest in the 1970s. It subsequently transformed into a densely populated urban slum settlement. Four valleys are included in the study site (approximately 0.5 km^2^).

Pau da Lima meets both the United Nations’, and the Brazilian Census Bureau’s criteria for an urban slum [4]. In the 2010 Census, the Brazilian Census Bureau created the unique term *aglomerado subnormal* to describe communities meeting the following criteria:

(1) illegal occupation of the land characterized by construction on the property of others or receipt of land title in the previous 10 years, and
(2) one of the following:
  - construction outside of existing municipal patterns, reflected by the presence of narrow and uneven roads, land parcels of inconsistent shape and size, and development not overseen by regulatory agencies, or
  - a general scarcity of public services [22].

### 2.2. Data Collection and Recruitment

In 2010, during enrollment of individuals from randomly selected households for a leptospirosis survey [23,24,25,26], researchers collaborated with the Pau da Lima Residents Association (RA) to conduct screening for diabetes mellitus (DM), hypertension, dyslipidemia, overweight, obesity, and smoking as a benefit to participants in the leptospirosis survey. All participants eighteen years and older were invited to visit the RA, while fasting, for NCD screening. At the RA they were interviewed using a standardized questionnaire, which collected information concerning self-reported height, weight, previous medical diagnoses of hypertension, DM, dyslipidemia, and current smoking status. Blood samples were collected in plain and sodium fluorite tubes and transported on the same day under refrigeration to the Faculty of Pharmacy at the Federal University of Bahia, where plasma glucose, triglycerides, and cholesterol were measured.

Collaborating with the community’s Health District Office, researchers arranged appointments with physicians at the local health facility for those with abnormal laboratory results, a prior diagnosis of hypertension but not on treatment, or a body-mass index (BMI) greater than 25 mg/kg^2^.

### 2.3. Clinical Definitions

The BMI of all participants was calculated and categorized according to World Health Organization (WHO) guidelines as normal weight (BMI ≥ 18.5 and < 25 kg/m^2^), overweight (BMI ≥ 25 and < 30 kg/m^2^), or obese (BMI ≥ 30 kg/m^2^) [27]. Participants reported whether or not they were active smokers at the time of the interview. In this study, DM was defined as a measured one-time non-fasting plasma glucose greater than 200 mg/dL, or a self-reported previous diagnosis of DM by a medical professional [28]. Hypertension definition was based on a self-report of prior diagnosis. 

Levels of low-density lipoprotein cholesterol (LDL-cholesterol), high-density lipoprotein cholesterol (HDL-cholesterol), and triglycerides were used to calculate total cholesterol per the Adult Treatment Panel (ATP) III report criteria. Dyslipidemia was defined as an LDL-cholesterol greater than 160 mg/dL, a total cholesterol above 240 mg/dL, or a self-reported previous diagnosis by a medical professional [29].

### 2.4. NCD Survey of Salvador

To investigate how the prevalences of NCDs in Pau da Lima population differed from the prevalences of NCDs in the overall population of Salvador, we compared the prevalences of NCDs among residents of Pau da Lima with the prevalences of NCDs among all residents of Salvador who participated in the 2010 Vigitel survey. This annual telephone survey interviews at least 2000 adults (18 and older) in every Brazilian state capital. It has been conducted by the Ministry of Health since 2006 with the express purpose of monitoring NCD burden in Brazil [30]. Sampling for Vigitel begins with the random selection of 5000 landlines per city. After screening for businesses, vacant homes, and inactive numbers, each selected number is called up to ten times, randomly selecting an individual in each household, until 2000 people have been interviewed. A standardized questionnaire is used to record respondents’ self-reports of previous medical diagnoses of NCDs (DM, hypertension, and, as of 2013, dyslipidemia) and self-reported weight, height, as well as other data used to evaluate risk for NCDs [31]. Age, sex, height, weight, DM, hypertension, and smoking data were excerpted for Salvador from the 2010 survey [32]. Vigitel began to collect data for dyslipidemia in 2013. As a result, 2013 data were used for comparisons in the present study [33]. 

There are three datasets described in this manuscript:

1. 2010 Pau da Lima Leptospirosis Survey—Randomly selected households in Pau da Lima, Bahia, Brazil [23,24,25,26].
2. 2010 Pau da Lima NCD Survey—Non-random subsample of leptospirosis survey respondents who chose to participate in supplemental NCD survey.
3. 2010 Vigitel Survey—Citywide telephone survey of residents of Salvador, Bahia, Brazil [32]. Due to the fact that dyslipidemia data began to be collected in Vigitel in 2013, only these data are included for this analysis [33].

### 2.5. Statistical Analyses

All analyses were performed in STATA Version 12.1 (Statacorp, College Station, TX, USA). To assess for selection bias between those who participated in the randomly selected leptospirosis study and those who chose to participate in the NCD survey, Pearson’s chi-squared test was used to assess for differences between leptospirosis cohort members who did and did not participate in NCD screening. 

Overall, age- and sex-specific prevalences for the surveyed population in the Pau da Lima community and the Vigitel survey were calculated as the proportion of participants who fulfilled clinical criteria for each condition. Measurements of glucose, triglyceride, cholesterol, and creatinine for those not fasting at least nine hours prior to blood draw were excluded from analyses [29]. One participant with invalid records of height and weight was also excluded. For the surveyed population in Pau da Lima and the surveyed population of Salvador, 95% confidence intervals (CIs) of unadjusted prevalences were estimated by non-parametric bootstrap; one thousand bootstrap samples were used to estimate the standard error.

Prevalences of DM, hypertension, overweight, obesity, smoking, and dyslipidemia from both the Vigitel survey and the Pau da Lima survey were age- and sex-standardized to the age and sex distributions of the city of Salvador reported in the 2010 Census, to allow direct comparisons of NCD prevalence [22]. Sex-specific prevalences of these NCDs were age-standardized, and the age-specific prevalences of all conditions were sex-standardized. The standard errors and 95% CI of adjusted rates were calculated [34]. All comparisons between Vigitel participants and Pau da Lima in the text refer to a comparison of these adjusted rates.

### 2.6. Human Participant Protection

The leptospirosis cohort study was approved by the ethics committee at the Oswaldo Cruz Foundation, the Brazilian National Committee for Ethics in Research (0021.0225.000-08), and the Yale Human Research Protection Program (1006006956). All participants provided written informed consent. The NCD and NCD risk factor survey data were not initially collected for research purposes, but as a service requested by community leaders and delivered by researchers at the Oswaldo Cruz Foundation. However, a 2014 protocol was approved by the ethics committee at the Oswaldo Cruz Foundation (11899212.1.0000.0040), granting a waiver for informed consent for the present use of these data.

## 3. Results

### 3.1. Demographic Distribution and Comparison of Surveyed Populations

Of 2331 participants in the leptospirosis cohort, 792 (34.0%) participated in the NCD survey. These residents were similar to the initial cohort that did not participate in NCD screening in terms of race and education, yet had more females, and was older and significantly wealthier (*p* <0.05) (Table 1). The Pau da Lima surveyed population had a mean age of 38.7 years and 511 (64.5%) were female.

### 3.2. Prevalence of Non-Communicable Diseases and Risk Factors

Seven hundred and seventy-three (97.6%) people had data available on all conditions (hypertension, DM, overweight or obese, obese, cholesterol, and smoking). The main reason for missing data was refusal to report (19, 2.4%). The number of residents reporting each condition varies due to differential rates of reporting and different numbers of individuals participating in laboratory testing.

#### 3.2.1. Diabetes Mellitus

**Figure 1:**
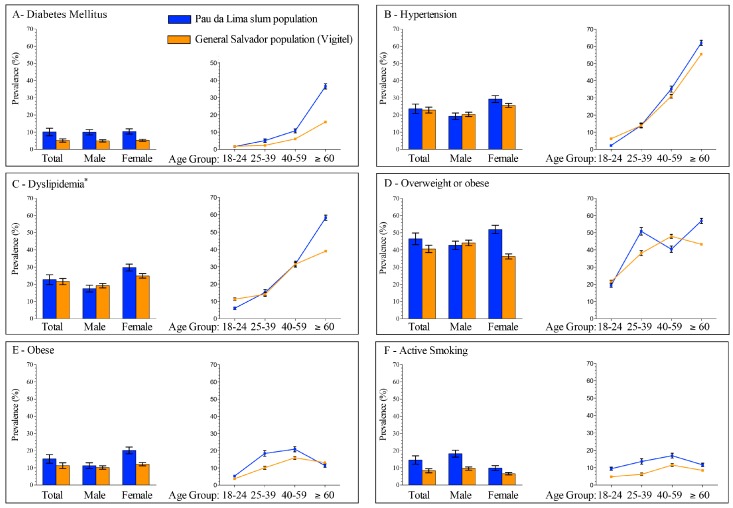
Estimated prevalences of non-communicable diseases and risk factors in Salvador, Brazil, 2010. Populations include Pau da Lima, Salvador, Brazil, 2010, and the 2010 Vigitel telephone survey of the general population of Salvador, Brazil. Total prevalence was adjusted for age and sex, sex-specific prevalence was adjusted for age, and age-specific prevalence was adjusted for sex. All adjustments were made to the age and sex profiles of Salvador in the 2010 Census. (**A**) Prevalence of diabetes mellitus; (**B**) prevalence of hypertension; (**C**) prevalence of dyslipidemia; (**D**) prevalence of overweight or obesity (body mass index (BMI) ≥ 25 kg/m^2^); (**E**) prevalence of obesity (BMI ≥ 30 kg/m^2^); and (**F**) prevalence of active smoking.

Of 783 participants in Pau da Lima, 69 (8.8%) reported a previous diagnosis of DM (56, 81.1%), or had a measured plasma glucose (PG) above 200 mg/dL at time of survey (13, 18.8%) (Table 2). The age- and sex-adjusted prevalence of DM was greater among residents of Pau da Lima (10.1%) than in the general population of the city of Salvador (5.2%) (Figure 1, Table S1).

#### 3.2.2. Hypertension

Of the 792 participants in Pau da Lima, 189 (23.8%) reported a previous diagnosis of hypertension (Table 2). The age- and sex-adjusted prevalence of hypertension was roughly the same among residents of Pau da Lima (23.6%) and in the general population of Salvador (22.9%) (Figure 1, Table S1), although women living in Pau da Lima had a higher age-adjusted prevalence of hypertension (29.3%) than women in the general population (25.6%). The prevalences of hypertension were also higher among those aged 40–59 (Pau da Lima (PDL): 35.2%; Vigitel: 30.9%) and those older than 60 (PDL: 62.1%; Vigitel: 55.5%) who lived in Pau da Lima compared with individuals of the same age in Salvador. 

#### 3.2.3. Dyslipidemia

Of 781 participants living in Pau da Lima, 183 (23.4%) had dyslipidemia (Table 2). In 2013, the prevalence of dyslipidemia in both populations was roughly the same (PDL: 21.4%; Vigitel: 21.5%) (Figure 1, Table S1).

#### 3.2.4. Overweight and Obese

Of 790 participants living in Pau da Lima, 387 (49.0%) were overweight or obese, and 137 (17.3%) were obese (Table 2). The overall adjusted prevalence of overweight and obesity was 46.5% among residents of Pau da Lima, compared to 40.6% of residents of Salvador. Fifteen percent (15.2%) of the population in Pau da Lima was obese, compared to only 11.1% of the population of Salvador (Figure 1, Table S1). The prevalences of overweight/obesity and obesity were higher among those living in Pau da Lima than among the population of Salvador, except among men, where there were roughly as many males in Pau da Lima who were overweight/obese (42.8%) as in the city of Salvador (44.1%).

In Pau da Lima, the prevalence of overweight or obesity, and obesity was higher among women (52.0% BMI ≥ 25 and 20.2% BMI ≥ 30) than among men (42.8% BMI ≥ 25 and 11.3% BMI ≥ 30) (Figure 1, Table S1).

#### 3.2.5. Smoking

Among the 792 participants living in Pau da Lima, 106 were smokers (13.4%) (Table 2). In Pau da Lima, almost one-fifth (18.2%) of males smoked, compared to only 9.9% of females (Figure 1, Table S1). More residents of Pau da Lima (14.5%) were smokers than in the city of Salvador (8.3%). The prevalence of smoking among men in Pau da Lima (18.2%) was almost double the prevalence of men who smoked in the general population of Salvador (9.5%). Those aged 18–24 in Pau da Lima also smoked at roughly twice the frequency (9.4%, 25–39: 13.6%) of those aged 18–24 in the city of Salvador (4.8%, 25–39: 6.2%).

## 4. Discussion

In this survey of a *favela* in Salvador, Brazil, we found higher prevalences of DM, smoking, and being overweight or obese than among the general population of Salvador. These high prevalences were mirrored in most age- and sex-specific estimates as well, with the largest disparity among Pau da Lima’s women. Women in Pau da Lima had a much higher BMI than men in their community, who were also more obese and overweight than the general population of Salvador. Women in Pau da Lima also had a higher prevalence of hypertension than men in Pau da Lima, or women in the city of Salvador. There were almost twice as many active smokers living in Pau da Lima than in the city of Salvador.

These results suggest that the prevalence of NCD in Pau da Lima is higher than in the city of Salvador, lending support to the hypothesis that slum residents have more NCD than their non-slum dwelling urban counterparts. The higher prevalence of NCD and cardiovascular risk factors in this slum population suggest that as it continues to age, it will be at greater risk for major cardiovascular events, such as heart attack or stroke, than the general population.

Very few studies have investigated the prevalence of NCD among residents of urban slums. A 2012 survey in a Lima, Peru community found lower prevalences of hypertension and DM, but similar prevalences of overweight, obesity, and smoking to our survey [35]. Serial cross-sectional surveys from a Kenyan slum in 2010 reported similar prevalences of hypertension, a lower prevalence of DM and a higher prevalence of overweight and obesity compared to the present study [7,16,36]. Several other studies in Kenyan and Nigerian communities have reported a lower prevalence of hypertension than among those living in Pau da Lima [8,10]. Importantly, none of these studies systematically compared the prevalence of these diseases in slums to the general population of the same city.

In 2011, the World Health Organization estimated that 14.1% of the Brazilian population smoked, 40.0% had elevated blood pressure, and 18.8% were obese [37]. These are higher than both Vigitel and the present survey. One explanation for this is differing estimation methodologies, but it could also be due to regional differences in disease prevalence; however, it is important to note that Vigitel is developed for and used by the Brazilian Ministry of Health, while WHO estimates may have alternative uses.

There were several limitations to this study. Both the Vigitel and the Pau da Lima surveys relied on self-reporting. In the Vigitel survey, every NCD was self-reported, whereas the Pau da Lima survey combined self-report and laboratory measures for DM and dyslipidemia, relying on self-report for hypertension, smoking, and overweight and obesity. Self-reporting can significantly underestimate NCD prevalence; however, this would likely be a differential bias, with wealthier (non-slum) residents more accurately reporting their disease status due to their higher level of education, and relatively better access to medical care.

The difference in disease burden between the general population of Salvador and the results of the Vigitel survey could be due to differences in survey methodology, but they may also be due to the well-documented and described physical and demographic differences between slum and non-slum areas. For example, residents of urban slums have limited access to healthcare, are less educated, and poorer compared with their non-slum dwelling urban counterparts [38,39]. This can lead to relatively worse health literacy or differences in nutrition. Further, drug-related violence, particularly in Brazil [40], limits access to health facilities, leaving fewer safe spaces for exercise and play. Many *favelas* are also located on rough or poor-quality terrain, such as mountain slopes, hindering travel and development of transportation infrastructure. Differential access to healthy food between these communities and wealthier regions also exists, given the elevated cost of fresh fruits and vegetables relative to more processed foods. 

Another limitation to this study is that it was done in only one community, and sampling was not random. Sampling in the initial leptospirosis cohort was random by household, but participants in the present survey were expected to report to the Pau da Lima RA. As such, it is possible that the study suffered from self-selection bias, but the Vigitel survey would also experience this same bias. Further, a large proportion of the world’s and Brazil’s slum populations live in similar tropical climates, with comparable rates of poverty and environmental degradation [4]. 

It is also important to highlight that citywide surveys conducted by telephone, such as Vigitel, likely do not generate viable sampling frames in slums. Only 40% of the 764 surveyed households in Pau da Lima had landlines in 2010. Consequently, the general population of Salvador represented by this survey may not have proportionally included slum residents, and would have likely underestimated the true burden of NCD in the overall population. On the other hand, because we did not compare the Pau da Lima population with the non-slum population of Salvador, but with the general Salvador population, the differences between the prevalences of NCD between slum and non-slum populations may even be conservatively low. There may also have been differential misclassification in identification of DM and dyslipidemia because poverty and education can confound the accuracy of self-reported medical information [41].

Further, we only used one PG reading to identify DM (cross-referencing with self-reported previous diagnosis). This would overestimate the DM prevalence were fasting incorrectly reported. However, in doing so, we identified 13 people who had not been told by a physician that they were diabetic. Active tobacco smoking was only reported as binomial (yes or no), not allowing for thorough definition as a risk factor. Cardiovascular risk is usually measured using scales such as the Framingham Risk Score, but due to lack of data availability, we were unable to calculate this score in these populations [42]. Further surveys that comprehensively collect data on cardiovascular risk must be done to evaluate risk for sequelae due to NCD. Despite this, self-reported data on weight, height, BMI [43], hypertension [44], and diabetes [45] have all been previously validated for epidemiologic research. Despite these drawbacks, these data, and the comparisons with Vigitel are very important, as they highlight a lack of surveillance and potentially-elevated NCD rates among an explicitly vulnerable population about which little is known: residents of urban slums. 

This differential NCD burden among slum residents imposes an enormous economic cost on the state, not only in terms of mounting healthcare costs from sequelae and mortality, but also in the form of an acute loss of labor from low-income workers and the informal economy when they become too sick to work. The informal economy is estimated to comprise roughly 40% of the economy in middle- and low-income countries, where the majority of the global slum population resides [46,47], and much of the construction, manufacturing, sales, and migrant workforce resides in these slums [48]. As the most common age demographic in Pau da Lima and other *favelas* is also the most active economically (those aged 25–60) [38], NCD sequelae would remove them from the workforce at an earlier age than their non-slum dwelling colleagues. Further, cardiovascular events are strongly associated with family impoverishment, usually affecting the most economically active individuals in a household [49], perpetuating multi-generational poverty. While efforts must be made to improve education and work opportunities in these communities, policy makers and health practitioners must enact concurrent efforts to limit morbidity and mortality from NCDs [47,50].

This work must also be situated within the broader context of social determinants of health and neighborhood-specific risk factors for disease. This eco-social theory highlights the role of structural and group-level conditions, such as education, wealth, class, sanitation and habitation infrastructure, and many more in the health-disease process. [51] The complex interactions between socio-geographic characteristics contribute to or prevent disease development, and mitigate or worsen disease outcomes. These can lead to worse health outcomes, especially in socioeconomically disadvantaged populations. Residence in a slum represents one of these structural conditions, neighborhood-specific risk, and this report is a direct effort to quantify and understand the problem in order to contribute to the development of solutions to these health inequities.

## 5. Conclusions

Long-term severe sequelae due to these NCDs can be prevented with early, direct social interventions that facilitate access to healthy food and exercise, while also offering competitive economic opportunities [52]. As the global burden of NCDs increases along with the number of people living in slums [4], researchers must explicitly quantify and compare disease risk in this vulnerable subpopulation with that of the general population. To contribute to policies that alleviate these disparities, researchers must begin to understand and describe the causal mechanisms responsible for these differential disease outcomes, and how living in a slum impacts this relationship.

## Figures and Tables

**Table 1:** Demographic and social characteristics of participants in the non-communicable disease (NCD) survey, compared to a random sample of residents of Pau da Lima *favela*, Salvador, Brazil, 2010.

		NCD Survey (*n*: 792)	Pau da Lima *Favela* Cohort (*n*: 1539)	*p*-Value
Female sex		511 (64.5%)	911 (58.1%)	<0.01
Age group (years)	18–24	128 (16.1%)	309 (19.7%)	0.01
	25–39	316 (39.9%)	649 (41.4%)	
	40–59	287 (36.2%)	506 (32.3%)	
	≥60	61 (7.7%)	105 (6.7%)	
Race	Black	436 (55.4%)	861 (55.1%)	0.84
	Mixed	300 (38.2%)	594 (38.0%)	
	Other	51 (6.5%)	107 (6.9%)	
Schooling (years)	0–3	156 (19.7%)	273 (17.4%)	0.50
	4–7	227 (28.6%)	484 (30.9%)	
	8–13	409 (51.6%)	812 (51.8%)	
Daily per-capita income (2010 US$)	<2.00	128 (17.5%)	338 (23.6%)	<0.01
	2.00–3.99	213 (29.1%)	518 (36.2%)	
	4.00–5.99	159 (21.8%)	311 (21.7%)	
	≥6.00	231 (31.6%)	264 (18.5%)	

**Table 2:** Unadjusted total, sex-, and age-specific prevalences of non-communicable diseases (NCDs) and NCD risk factors in the community of Pau da Lima, Salvador, Brazil, 2010.

	Total		Sex				Age							
			Male		Female		18–24		25–39		40–59		≥60	
	(%)	95% CI	(%)	95% CI	(%)	95% CI	(%)	95% CI	(%)	95% CI	(%)	95% CI	(%)	95% CI
Diabetes mellitus	8.8	6.8, 10.8	9.8	6.4, 13.2	8.3	5.9, 10.7	1.6	0, 3.5	4.8	2.4, 7.2	10.6	7.1, 14.1	36.7	24.6, 48.7
Hypertension	23.8	20.8, 28.2	19.9	15.3, 24.6	26.0	22.3, 29.8	2.3	0.0, 4.7	14.2	10.3, 18.2	35.9	30.2, 41.6	62.3	49.8, 74.8
Dyslipidemia	23.4	20.4, 26.5	17.2	12.8, 21.6	26.8	22.9, 30.7	6.3	2.2, 10.5	14.7	10.7, 18.7	33.1	27.8, 38.4	59.0	46.8, 71.3
Overweight or obese ^1^	49.0	45.5, 52.5	43.4	37.6, 49.2	52.1	47.8, 56.5	23.5	13.8, 33.2	51.0	44.0, 58.1	60.1	54.0, 67.6	48.7	33.1, 64.4
Obese ^2^	17.3	14.8, 19.9	10.7	7.2, 14.2	21.0	47.8, 56.5	5.9	0.3, 11.4	13.4	8.7, 18.1	12.4	7.7, 17.1	12.8	2.4, 23.2
Active smoker	13.4	11.1, 15.7	18.1	13.7, 22.6	10.8	8.2, 13.5	8.6	3.8, 13.4	12.7	8.9, 16.4	16.7	12.3–21.1	11.5	3.3–19.6

^1^ Overweight or obese defined as a BMI ≥ 25 kg/m^2^; ^2^ Obese defined as a BMI ≥ 30 kg/m^2^.

## Supplementary Materials

The following are available online at http://www.mdpi.com/2414-6366/2/3/47/s1, Table S1: Age- and sex-adjusted total, sex-adjusted age-specific, and age-adjusted sex-specific prevalences of non-communicable diseases and risk factors in Pau da Lima (PDL), Salvador, Brazil, 2010 and the 2010 Vigitel telephone survey in Salvador.
