# Supplementary material for: Differences in the Prevalence of Non-Communicable Disease between Slum Dwellers and the General Population in a Large Urban Area in Brazil
Source: Trop Med Infect Dis. 2017 Sep 16;2(3):47. doi: 10.3390/tropicalmed2030047 (PMC6082112; doi:10.3390/tropicalmed2030047)
Supplement: Supplementary file 1 [file tropicalmed-02-00047-s001.pdf]

Table S1. Age- and sex-adjusted total, sex-adjusted age-specific and age-adjusted sex-specific prevalences of non-communicable diseases and risk factors in Pau da Lima (PDL), Salvador, Brazil, 2010 and the 2010 Vigitel telephone survey in Salvador.

	Pop.	Total		Sex				Age							
				Male		Female		18–24		25–39		40–59		≥60	
		(%)	95% CI	(%)	95% CI	(%)	95% CI	(%)	95% CI	(%)	95% CI	(%)	95% CI	(%)	95% CI
Diabetes mellitus	PDL	10.1	7.9, 12.3	10.0	8.4, 11.5	10.45	8.9, 12.0	1.7	1.3, 2.1	5.1	4.1, 6.1	10.8	9.6, 11.9	36.5	35.0, 38.0
	Vigitel	5.2	4.2, 6.1	5.0	4.3, 5.6	5.3	4.7, 5.9	1.8	1.6, 2.1	2.4	1.9, 2.8	6.1	5.5, 6.7	15.9	15.5, 16.4
Hypertension	PDL	23.6	20.9, 26.4	19.3	17.4, 21.2	29.3	27.3, 31.3	2.3	1.9, 2.7	13.9	12.5, 15.4	35.2	33.4, 36.9	62.1	60.6, 63.6
	Vigitel	22.9	21.2, 24.6	20.3	19.1, 21.6	25.6	24.4, 26.8	6.2	5.7, 6.7	13.9	12.9, 14.9	30.9	29.9, 32.0	55.5	54.8, 56.1
Dyslipidemia [1]	PDL	22.7	19.8, 25.5	17.5	15.5. 19.5	29.7	27.7, 31.7	6.0	5.3, 6.7	15.1	13.4, 16.7	31.5	29.7, 33.2	58.3	56.8, 59.8
	Vigitel	21.5	19.7, 23.4	19.2	17.9, 20.5	24.9	23.6, 26.2	11.3	10.5, 12	14.1	12.9, 15.2	31.5	30.4, 32.6	39.0	38.5, 39.6
Overweight or obesity[2]	PDL	46.5	43.1, 49.9	42.8	40.3, 45.2	52.0	49.6, 54.4	19.4	18.3, 20.6	50.9	48.7, 53.1	40.5	38.6, 42.3	56.9	55.4, 58.4
	Vigitel	40.6	38.5, 42.8	44.1	42.5, 45.7	36.3	34.8, 37.7	21.6	20.8, 22.4	38.1	36.7, 39.6	47.9	46.8, 49.1	43.3	42.7, 44
Obesity [3]	PDL	15.2	12.7, 17.7	11.3	9.7, 12.9	20.2	18.2, 22.1	5.3	4.6, 5.9	18.4	16.8, 20.1	20.9	19.4, 22.4	11.4	10.4, 12.4
	Vigitel	11.1	9.3, 12.9	10.2	9.2, 11.2	12.1	11.2, 13.1	3.7	3.3, 4.1	10.1	9.2, 11	15.9	15.0, 16.7	13.1	12.7, 13.5
Active smoker	PDL	14.5	12.1, 17	18.2	16.2, 20.2	9.9	8.5, 11.3	9.4	8.5, 10.3	13.6	12, 15.1	16.9	15.5, 18.3	11.6	10.6, 12.6
	Vigitel	8.3	7.1, 9.5	9.5	8.6, 10.5	6.6	5.9, 7.3	4.8	4.4, 5.2	6.2	5.4, 6.9	11.5	10.7, 12.3	8.4	8.1, 8.8

[1] Dyslipidemia estimates from 2013 used, not reported in Vigitel until 2013. [2] Overweight or obesity defined as a BMI ≥25 kg/m^2. [3] Obesity defined as a BMI ≥30 kg/m^2.
